# Dried blood spot cards: A reliable sampling method to detect human antibodies against rabies virus

**DOI:** 10.1371/journal.pntd.0008784

**Published:** 2020-10-13

**Authors:** Laura Doornekamp, Carmen W. E. Embregts, Georgina I. Aron, Simone Goeijenbier, David A. M. C. van de Vijver, Eric C. M. van Gorp, Corine H. GeurtsvanKessel

**Affiliations:** 1 Department of Viroscience, Erasmus MC, University Medical Center Rotterdam, WHO Collaborating Centre–Emerging Viral Infections, Rotterdam, the Netherlands; 2 Travel Clinic, Erasmus MC, University Medical Center Rotterdam, Rotterdam, the Netherlands; 3 Department of Internal Medicine, Erasmus MC, University Medical Center Rotterdam, Rotterdam, the Netherlands; University of Surrey, UNITED KINGDOM

## Abstract

**Background:**

Although preventable by vaccination for more than a century, rabies virus still causes numerous fatalities every year. To determine antibody levels in humans, blood collected with a finger prick and applied on dried blood spot (DBS) cards is an alternative for venipuncture. The use of DBS is specifically valuable in remote areas, as it is easy to perform, store and transport. Therefore, the technique is frequently used for epidemiological studies of tropical diseases. Up to present, determination of rabies virus antibody levels on human DBS has not been validated.

**Methodology/Principal findings:**

We evaluated the use of human DBS for rabies serology and analyzed 99 pre- or post-vaccination serum and DBS samples with a fluorescent antibody virus neutralization test (FAVNt), which is the gold standard to detect protective antibody levels, and a Bio-Rad Platelia Rabies II ELISA. Sensitivity and specificity of DBS eluates tested with the FAVNt were 97% and 92%, respectively and 87% and 96% when tested with the Platelia-II ELISA. Antibody levels measured in serum with the FAVNt, correlated best with antibody levels measured in DBS with the FAVNt (R = 0.88).

**Conclusions/Significance:**

This is the first study that applies DBS for reliable detection of human antibodies against rabies virus. Both the FAVNt and Platelia-II ELISA demonstrate an acceptable performance on DBS, providing opportunities for rabies serology in remote areas. This technique could drastically ease studies evaluating (novel) rabies vaccination strategies and monitoring persisting immunity in humans at risk, living in rabies endemic regions.

## Author summary

Rabies is a nearly 100% fatal disease in humans. However, available vaccines are effective in preventing rabies infection. To investigate if a person is protected against rabies, rabies virus neutralizing antibody levels in the blood are determined. The World Health Organization defines protective immunity as a rabies virus antibody concentration of at least 0.5 IU/ml detected in serum using a virus neutralization test. Yet, in remote areas serum may be rather difficult to collect, process and transport. Whole blood collected with a finger prick and applied on filter paper cards, also known as dried blood spots (DBS), are an easier alternative. This collection method is frequently used for serology of several tropical infectious diseases, but never studied for rabies serology in humans. Therefore, we compared antibody levels measured in serum with those measured in DBS eluates, using the gold standard FAVNt and related it to another commonly used test for human rabies serology, the Platelia-II ELISA. We found that both assays had a good performance on DBS eluates. The reported high specificities provide confidence that unprotected individuals will rarely be missed. Therefore, the DBS is a promising sampling technique for evaluations of vaccination strategies and monitoring persisting immunity after vaccination in populations at risk for rabies.

## Introduction

Despite the availability of safe and effective vaccines, rabies virus (RABV) causes approximately 59.000 fatal infections every year [1]. Pre-exposure prophylaxis (PrEP) is indicated when the risk of exposure to rabies is high and can go unnoticed (e.g. occupational exposure); when fast access to post-exposure prophylaxis is limited (e.g. in remote areas); and when it is difficult to control rabies in the animal reservoir [2, 3]. For healthy individuals PrEP currently consists of two vaccinations with an interval of seven days, after which an individual is normally considered to be lifelong protected [3, 4]. However, in individuals with occupational exposure, protective immunity should be checked every one to two years [3]. Protective immunity is defined by the World Health Organization (WHO) as a rabies virus neutralizing antibody (RVNA) concentration ≥0.5 IU/ml detected in serum using the rapid fluorescent focus inhibition test (RFFIT) [5] or the fluorescent antibody virus neutralization test (FAVNt) [1, 6]. These gold standard assays are reliable but expensive, time-consuming and require qualified biosafety level 3 laboratories with trained employees. An enzyme-linked immunosorbent assay (ELISA) is a less demanding alternative for human rabies serology and is therefore easier to implement in low-resource settings [7]. In previous human studies the Bio-Rad Platelia Rabies II ELISA, measuring binding of antibodies to the virus’ glycoprotein, showed a good performance when compared to the FAVNt [8]. Nevertheless the ELISA should be considered as a surrogate to determine immunity against rabies, given that it measures all antibodies that bind the RABV glycoprotein, in contrast to the FAVNt that specifically measures functional virus neutralizing antibodies.

Both FAVNt and ELISA are usually performed on serum, but blood collected with a finger prick on dried blood spot (DBS) cards can be a valuable alternative, specifically when specimens are collected in remote areas. DBS cards can be easily obtained and transported, facilitating on-site sampling [9]. DBS cards have been used for decades for various purposes, from early neonatal screenings for metabolic diseases [10] to molecular or serological diagnosis of infectious diseases and therapy monitoring [9, 11, 12], but it has not yet been applied for detection of RVNA in human populations [13]. The only reported experience with rabies serological assays on filter paper cards, is a study performed in foxes and raccoons. Although in this study thicker Mini Trans-Blot Filter Paper cards were used, promising results were reported [14].

The ease of DBS sampling makes it an appropriate tool in monitoring immunity against rabies virus, serosurveillance or other types of studies in resource-limited areas in support of the global agenda to end human rabies transmitted by dogs [3]. The minimal invasiveness of DBS cards eases the collection of samples in settings outside medical care facilities, which is crucial as 80 percent of human rabies infections occur in rural areas [15]. Furthermore, it is a commonly used technique in young children. Forty percent of the victims are under 15 years of age [15], in whom venipuncture can be difficult to perform.

In this study, we evaluate the performance of both the FAVNt and the Platelia-II ELISA on DBS eluates in comparison to serum samples and present promising results.

## Methods

### Sample collection

Forty-eight students that were rabies vaccinated between 2015–2018 were selected from the Vaccination Cohort (COVA) Biobank study that was carried out at the Travel Clinic of Erasmus MC, Rotterdam, the Netherlands. As the rabies vaccination guidelines for travelers changed during the study period, either three or two intramuscular vaccinations (with Verorab or Rabipur) were given on day 0, 7 (and 21–28) [4]. Upon informed consent for participation in the COVA biobank, students were sampled pre- and (1–30 months) post-vaccination, resulting in 99 paired venipuncture and DBS (blood directly aspirated from the serum separating tube and applied on a Whatman Protein Saver 903 Card) samples. After two to three hours drying at room temperature, each DBS card was stored in a foil-barrier zip lock bag with a desiccant sachet and kept for a maximum of two weeks at room temperature [16]. Serum was centrifuged and kept at four degrees Celsius for a maximum of four weeks. Serum and DBS cards were stored respectively at -20 or -80 degrees Celsius until testing, because of the prolonged time period between collection and testing [17].

### FAVNt

Elution of DBS in order to reach the highest serum concentration was optimized during pilot studies and resulted in an elution of 4 DBS card punches in 520 microliter PBS with 2% FBS during the study [16, 18]. DBS punches contained 26 microliter serum each [18], resulting in a serum dilution of 1:6 (104 μl serum in 520 μl PBS+2%FBS). Eluates were incubated overnight at four degrees Celsius on a rotating device [18]. The next day, eluates were heat-inactivated for 60 minutes at 56 degrees Celsius. Eluates were centrifuged at 3000 rpm for five minutes to remove any debris that can be formed during elution [16], before being processed for the FAVNt as described by Cliquet et al, 1998 [6]. Serum samples were heat-inactivated prior to use in the FAVNt and processed simultaneously with the eluates [6]. Microtiter plates were read blinded. The lower limit of detection of serum RVNA with the FAVNt was 0.06 IU/ml, which resulted in a lower detection limit of 0.36 IU/ml of the DBS eluates due to the dilution factor. The upper limit of quantification is 13.77 IU/ml.

### Platelia-II ELISA

During our pilot studies, we have found that the elution protocol that was used for the FAVNt, resulted in background signal in the Platelia-II ELISA. Therefore, a separate, optimized protocol was used for Platelia-II ELISA. First, individual punches were eluated in 858 microliter PBS with Surfact-Amps Detergent Sampler (to result in a starting serum dilution of 1:34), while shaking overnight at four degrees Celsius. The next day, eluates were incubated for 90 minutes with 1716 microliter Blocker Blotto Blocking Buffer (to result in a final serum dilution of 1:100, which is the recommended dilution for the ELISA) and were centrifuged afterwards. Microplates were pre-treated with the blocking buffer for 90 minutes to avoid aspecific binding [19]. Thereafter, the Platelia-II ELISA was performed quantitatively following the protocol of the manufacturer using the threshold of positivity set on 0.5 IU/ml, as described by Wasniewski et al [13]. Serial dilutions of the positive control serum (containing 4 IU/ml) were included for quantification of the results. Additional serial dilutions in sample buffer of 1:500 and 1:1000 for each serum and DBS sample allowed us to measure titer values up to 40 IU/ml.

### Statistical methods

Based on a previous vaccination study in students [20] and on the composition of our study cohort (19 pre-vaccination samples and 80 post-vaccination samples taken 1–30 months post-vaccination), the expected seroprevalence of antibodies was 80 percent, which is comparable to situations in which monitoring or evaluation of vaccination strategies are performed [20]. In these circumstances, a specificity of at least 90 percent will likely result in an acceptable positive predictive value. A sample size calculation was performed with a power of at least 0.80 and a p-value smaller than 0.05, and resulted in a sample size of approximately 100 [21]. Coefficients of variance (CV) were calculated to define the repeatability and a value <20 percent was considered as acceptable. GraphPad Prism version 5.0 for Windows (GraphPad Software, La Jolla California USA, www.graphpad.com) was used for statistical analysis and to create figures.

### Ethics

This study was performed on samples from the Vaccination Cohort (COVA) biobank. Due to the biobank format, the work has been exempted from medical ethical approval requirements by the medical ethical research committee of the Erasmus MC Rotterdam, the Netherlands (MEC-2014-398). Written informed consent has been obtained from every participant and data was analyzed anonymously. The study has been conducted according to the principles of the declaration of Helsinki (59th version, WMA General Assembly, Seoul, October 2008).

## Results

The FAVNt and Platelia-II ELISA results on DBS eluates were compared to the gold standard, which is serum tested by FAVNt (serum-FAVNt), and applying the WHO recommended threshold of protection (0.5 IU/ml). When using the serum-FAVNt, the RVNA titers of the 80 post-vaccination serum samples tested ranged from 0.17 to 13.77 IU/ml (S1 Data), with an average of 3.38 and a standard deviation of 3.23 IU/ml. The RVNA titers of 19 pre-vaccination serum samples were all 0.06 IU/ml. The FAVNt on the paired DBS eluates (DBS-FAVNt) showed a sensitivity and specificity of respectively 97.3 percent (95% CI 90.6–99.7) and 92.0 percent (95% CI 74.0–99.0) when compared to serum-FAVNt. Only two false positives (with RVNA concentrations of 0.60 and 0.60 IU/ml) and 2 false negatives (0.36 and 0.42 IU/ml) were found.

The DBS-ELISA showed a sensitivity of 86.5 percent (95% CI 76.6–93.3) and a specificity of 96.0 percent (95% CI 79.7–99.9) compared to the gold standard serum-FAVNt. The sensitivity increased to 91.6 percent (95% CI 82.5–96.8) when the DBS-ELISA was compared with serum-ELISA. Moreover, the specificity increased to 100 percent (95% CI 87.7–100.0).

The intra-assay variation of serum-FAVNt in our WHO reference laboratory was assessed during yearly WHO proficiency testing. With a mean CV of 2.9 percent (WHO proficiency report 2019 [22], n = 14, tested in triplicate), the assay had an excellent performance. To assess repeatability, five DBS samples were eluated and tested in triplicate in separate plates for both the FAVNt and Platelia-II-ELISA. The average CV for DBS-FAVNt was 11.6 percent (RVNA titers range 0.5–7.92 IU/ml). For the DBS-ELISA the mean CV was 18.1 percent. In serum, the Platelia-II-ELISA manual reported a reproducibility of less than 10 percent.

**Fig 1:**
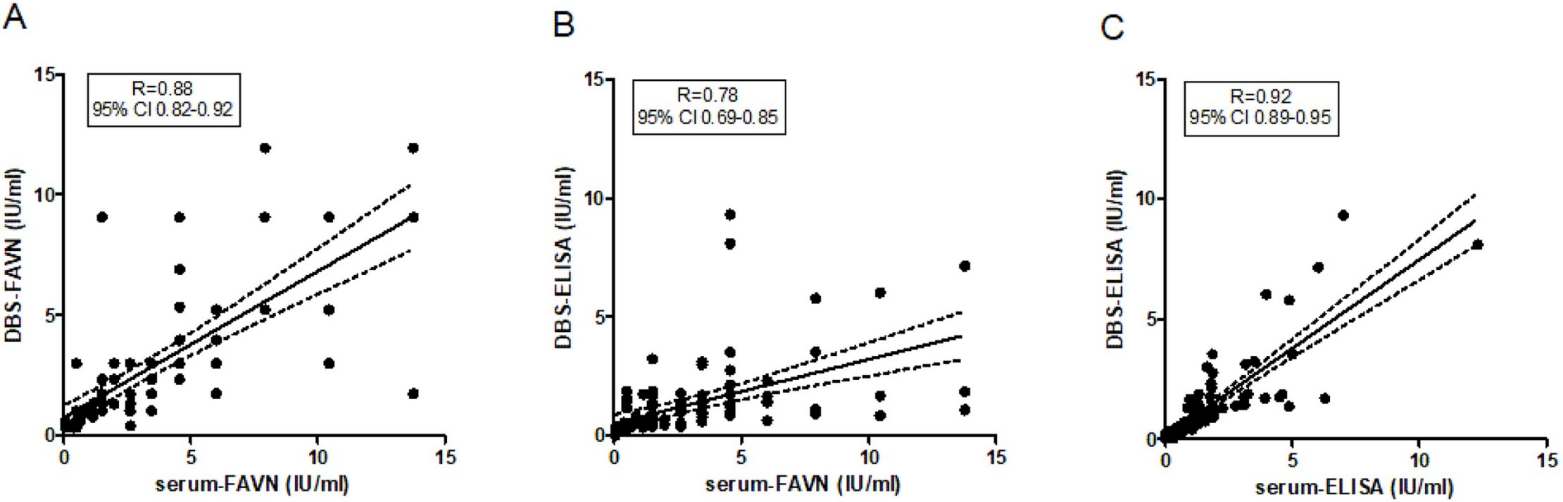
Correlation of quantitative results of the FAVNt and the Platelia-II ELISA performed on DBS and serum. Panel A shows the correlation between the RVNA titer of DBS-FAVNt and the RVNA titer of undiluted serum measured with the FAVNt. Panel B shows the antibody titer of DBS-ELISA compared to the RVNA titer of undiluted serum measured with the FAVNt. Panel C shows the antibody titer of DBS-ELISA compared to the antibody titer of serum-ELISA. R represents the Spearman correlation coefficient. CI is confidence interval. Dashed lines represent the of 5% confidence bands of the best fit line (represented by solid lines).

Because the concentration of RABV (neutralizing) antibodies is being correlated to the duration of protection [23], we investigated the quantitative performance of both assays on DBS eluates. Total IgG levels in 5 eluates were comparable with those in matched serum samples (1:6 diluted), with a mean CV value of 9 percent. The antibody concentrations measured in DBS eluates and sera correlated significantly when measured with either FAVNt or Platelia-II ELISA (Fig 1). However, the DBS-FAVNt results corresponded better to serum-FAVNt (Spearman R = 0.88, Fig 1A) than the DBS-ELISA (Spearman R = 0.78, Fig 1B). The titers in the DBS-ELISA correlated best with serum-ELISA titers (Spearman R = 0.92, Fig 1C).

**Fig 2:**
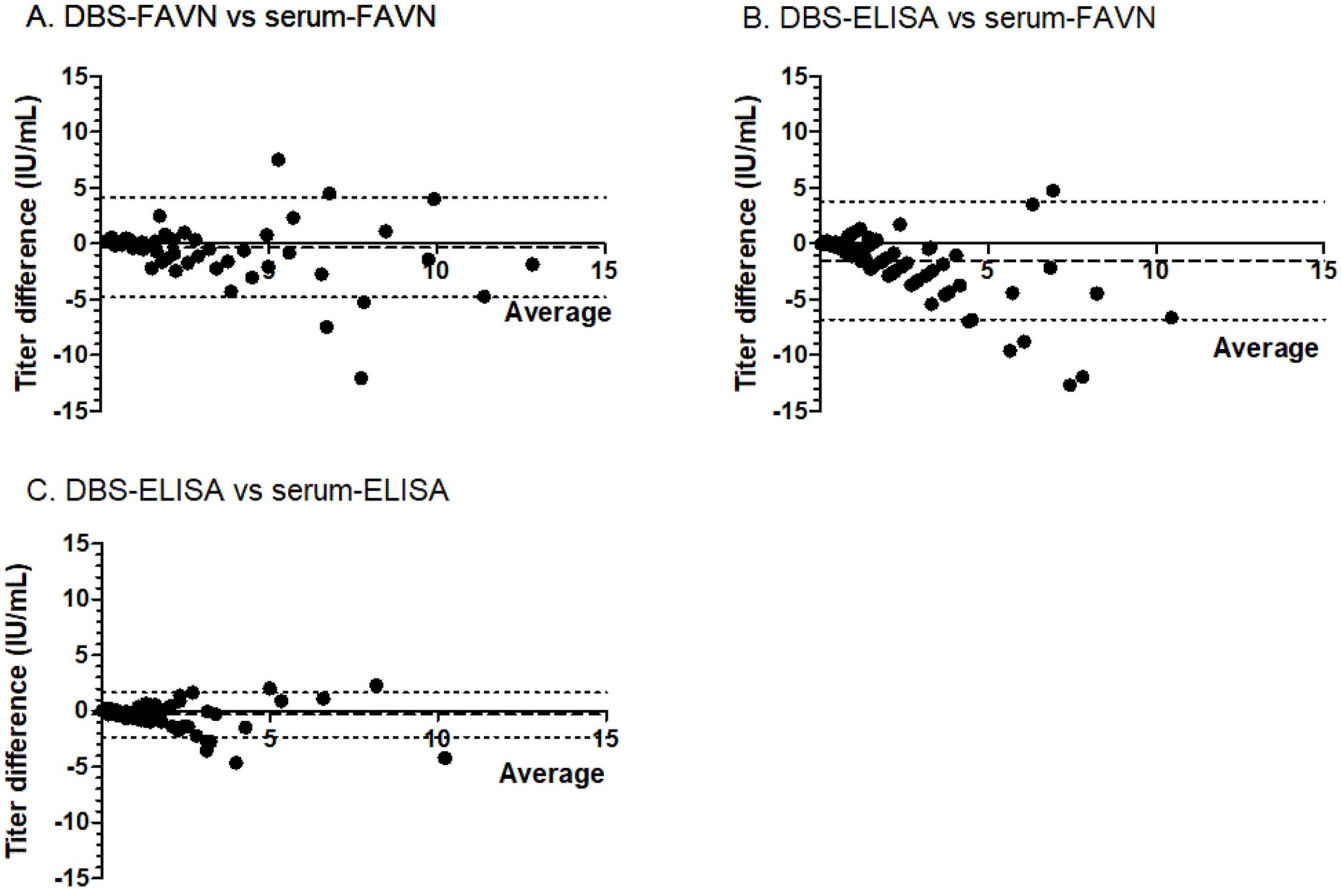
Bland-Altman plots of the mean titer of DBS and serum plotted against the difference between both values. Panel A shows the average antibody titer (IU/ml) of the DBS and sera measured with the FAVNt on the x-axis and the value (IU/ml) of the DBS minus the value of the serum on the y-axis. Panel B shows the average value of the DBS-ELISA and the serum-FAVNt on the x-axis and the value of the DBS-ELISA minus the value of the serum-FAVNt on the y-axis. Panel C shows the mean antibody level of the DBS and sera measured with the Platelia-II-ELISA on the x-axis and the value of the DBS minus the value of the serum on the y-axis. The long-dashed lines close to y = 0 represent the mean bias, the short-dashed lines represent the 95% limits of agreement (mean bias ±1.96 times the SD).

Finally, the agreement between the quantitative results on DBS and serum-FAVNt was determined by Bland-Altman plots (Fig 2). RABV antibody concentrations were on average 0.33 IU/ml lower in DBS-FAVNt versus serum-FAVNt (Fig 2A), and 1.48 IU/ml lower in DBS-ELISA versus serum-FAVNt (Fig 2B). DBS-ELISA antibody levels were on average 0.32 IU/ml lower than in serum-ELISA (Fig 2C). All three plots however show that the assays on DBS eluates were reliable when RVNA concentrations are low (<3.0 IU/ml).

## Discussion

In this study, we show for the first time that blood collection on DBS can be used for the detection of either neutralizing or binding antibodies against rabies virus in humans. When compared to the WHO gold standard in rabies serology, in which RVNA are measured in serum, the use of DBS eluates in the FAVNt performs best. The high sensitivity assures that more than 97 percent of the individuals who are protected against rabies will be detected, whereas 92 percent of the negatives will be found. Besides the detection of protective immunity in populations at high risk for (unnoticed) exposure, like veterinarians, medical and nursing hospital staff and laboratory workers [3, 24], DBS can be applied as a tool in monitoring immunity against rabies virus in resource-limited, endemic areas. Given that RABV has a case-fatality rate approaching 100%, reliably monitoring of post-vaccination titers is of utmost importance. Both the DBS-FAVNt and the DBS-ELISA have a high specificity, and can thus be used to determine which individuals at risk are in need of revaccination. Although the DBS-ELISA performs better than the DBS-FAVNt in terms of specificity, and could therefore be used as large scale screening method, the FAVNt with its complementary high sensitivity, prevents unnecessary administration of booster vaccinations.

The most probable reason for a higher correspondence between the DBS-FAVNt and serum-FAVNt than the DBS-ELISA and the serum-FAVNt, lies in the fact that both assays measure antibodies with different characteristics. The Platelia-II ELISA detects all antibodies that bind rabies glycoprotein, whereas the FAVNt measures the fraction of functional, neutralizing RABV antibodies [1]. The fact that RVNA are directed against the rabies glycoprotein, the protein that contributes to protection [25], nevertheless makes the Platelia-II ELISA a suitable alternative.

The Bland-Altmann plots demonstrate largest deviations at high (>3.0 IU/L) RVNA levels. This will likely not affect decision making in the administration of booster vaccines as these are given once titers reach 0.5 IU/L, an area in which the assay is very reliable. The fact that the levels of RABV antibodies are slightly lower in DBS compared to serum in all three comparisons, is likely due to a minor overestimation of the amount of serum in certain DBS eluates. This can also explain the few false negative results in DBS-FAVNt. A DBS elution of 1:6 was the lowest feasible elution in our pilot studies, which results in a lower limit of detection of DBS-FAVNt of 0.36 IU/ml, close to the recommended threshold of protection (0.5 IU/ml). One could consider to choose a slightly higher test cut-off when using DBS in the FAVNt, as the two false positive results were both only 0.6 IU/ml. In case a RVNA titer measured in DBS is considered negative when being equal to or lower than 0.6 IU/ml, the specificity will increase up to 100 percent. As a consequence, the sensitivity will slightly decrease and become 94.6 percent, which is still acceptable.

The repeatability of the FAVNt (mean CV = 3%) and the Platelia-II ELISA (reproducibility <10%) when testing sera is high. When using DBS eluates in the assays, the mean CV increases to 12 for DBS-FAVNt and to 18 for DBS-ELISA. Although we have estimated the volume of blood on the DBS by comparing IgG levels to serum samples, the amount of blood on the DBS cards can be an important factor in the variation of the assays. Therefore, adequate instructions when performing a finger prick and filling DBS cards, is crucial. In addition, cautious interpretation of antibody levels obtained by the use of DBS cards is of importance in which the laboratory assay variation should be taken into account and a cut-off should be determined carefully prior to application of the assays.

Although this is the first report in which the use of DBS is applied in human rabies serology, DBS were previously studied in other viral infections [9]. Studies are difficult to compare, due to the specific characteristics of antibodies and the differences in serological assays and elution protocols. A successful example of the use of DBS is in early detection of hepatitis B and C infections in resource-limited countries, which is now possible and recommended by the WHO. This advances treatment initiation and may finally prevent chronic complications in endemic regions [11, 26]. A quantitative meta-analysis in which the performance of DBS was assessed reported pooled estimates of a sensitivity of 98 percent and specificity of 99 percent. Other studies showed the use of DBS for the detection of measles-specific IgG concentrations [27], and anti-hepatitis A virus antibodies [28]. The latter showed that measuring antibodies in post-vaccination samples decreased the sensitivity to 77–98 percent (depending on the time elapsed post-vaccination) when compared to post-infection samples, suggesting that the assay is less sensitive in the lower range, which we did not observe. The application of rabies serology differs largely from testing high levels of naturally acquired, neutralizing antibodies against measles and hepatitis A virus. In rabies serology the aim is to detect protected individuals, mostly upon vaccination, although DBS sampling can also be used to further investigate incidental natural, non-lethal rabies exposure. Whereas rabies is generally seen as an inevitable fatal disease, this idea is challenged by some studies that found rabies antibodies in serum of unvaccinated populations living in endemic areas [29]. The studies are limited—as populations with high exposure to possible rabies-infected wildlife are often hard to reach—and heterogeneous due to the variance in serological assays and used cut-offs [30]. DBS sampling provides solutions for both issues, and could therefore contribute to the understanding of the immunopathology of rabies in seropositive populations. Here, the DBS-FAVNt has the preference, because the high sensitivity assures that most seropositive individuals will be detected.

The WHO strives to end human deaths from dog-mediated rabies by 2030 [15]. Elimination programs are usually evaluated by seroepidemiology and disease surveillance data. As rabies cases are often not diagnosed in endemic areas, disease surveillance is very insensitive to monitor elimination programs for rabies. Seroepidemiological studies could provide insight in vaccination status of risk groups and serosurveys could determine risk groups with suboptimal protection [24, 31]. Furthermore, serology is important as an endpoint in clinical trials of newly developed vaccines, different vaccination schedules or other administration routes. These developments are ongoing in rabies prevention strategies, as the availability and costs of the currently used rabies vaccines in endemic regions cause difficulties [1]. Determination of either rabies neutralizing or binding antibody levels–performed on easily obtained DBS–can be very supportive in large-scale evaluation of new vaccination strategies in endemic regions.

In conclusion, we demonstrate that DBS sampling provides accurate results in RABV-vaccinated and non-vaccinated adult travelers from a European country. As blood collection on DBS eases sampling and transportation, it is a promising alternative for rabies serology in children and in remote settings. Performance needs to be evaluated specifically in endemic regions and risk populations. With these promising results, DBS provides opportunities for evaluation of rabies vaccination trials and monitoring of persistence of immunity against rabies in humans in high-risk areas.

## Supporting information

S1 Data Dataset rabies serology results from the FAVNt and Platelia-II ELISA on serum and DBS. (XLSX)
